# Action and Traction: Cytoskeletal Control of Receptor Triggering at the Immunological Synapse

**DOI:** 10.3389/fimmu.2016.00068

**Published:** 2016-03-07

**Authors:** William A. Comrie, Janis K. Burkhardt

**Affiliations:** ^1^Department of Pathology and Laboratory Medicine, Children’s Hospital of Philadelphia, Perelman School of Medicine, University of Pennsylvania, Philadelphia, PA, USA

**Keywords:** immunological synapse, actin, cytoskeleton, mechanotransduction, integrin, T cell receptor, adhesion, costimulation

## Abstract

It is well known that F-actin dynamics drive the micron-scale cell shape changes required for migration and immunological synapse (IS) formation. In addition, recent evidence points to a more intimate role for the actin cytoskeleton in promoting T cell activation. Mechanotransduction, the conversion of mechanical input into intracellular biochemical changes, is thought to play a critical role in several aspects of immunoreceptor triggering and downstream signal transduction. Multiple molecules associated with signaling events at the IS have been shown to respond to physical force, including the TCR, costimulatory molecules, adhesion molecules, and several downstream adapters. In at least some cases, it is clear that the relevant forces are exerted by dynamics of the T cell actomyosin cytoskeleton. Interestingly, there is evidence that the cytoskeleton of the antigen-presenting cell also plays an active role in T cell activation, by countering the molecular forces exerted by the T cell at the IS. Since actin polymerization is itself driven by TCR and costimulatory signaling pathways, a complex relationship exists between actin dynamics and receptor activation. This review will focus on recent advances in our understanding of the mechanosensitive aspects of T cell activation, paying specific attention to how F-actin-directed forces applied from both sides of the IS fit into current models of receptor triggering and activation.

## Introduction

During their circulation through blood, lymphoid tissues, and peripheral sites of inflammation, T cells encounter and respond to a variety of environmental stimuli. Several of these responses are dependent on the application of external forces. A good example of this is the shear flow-induced activation of cell adhesion molecules during the slow rolling and firm adhesion steps of diapedesis, the process that brings cells from the bloodstream into tissues. Following diapedesis, T cells generate internal forces that drive their migration through the tissue stroma, in search of antigen-presenting cells (APCs) bearing major histocompatibility complex molecules loaded with their cognate peptides (pMHC). When T cells recognize these APCs, a specialized adhesive contact known as the immunological synapse (IS) is formed. The IS promotes sustained T cell/APC interactions and serves as a platform for exchange of information between the two cells. As with diapedesis and migration, T cell/APC adhesion and signal transduction at the IS depend on physical forces exerted by actin cytoskeletal dynamics. As detailed further below, actin-dependent protrusive forces drive close apposition of the two cells, bringing receptors on the T cell in contact with ligands on the APC. In addition, some IS-associated signaling molecules are physically linked to actin filaments; forces exerted on these molecules by the actin network result in conformational changes needed for full T cell activation.

Signaling at the IS takes place in dynamic microclusters containing surface receptors and downstream signaling molecules. These microclusters form at the periphery of the IS, within a region rich in branched actin filaments, reminiscent of the lamellipodium found at the leading edge of a migrating cell, and then move toward the center of the IS in parallel with centripetal flow of the actomyosin network (1). Importantly, ongoing actin flow is needed to sustain TCR signaling; if flow is arrested, intracellular Ca^2+^ levels drop, and early signaling intermediates are rapidly dephosphorylated (2). Although the signaling events that direct F-actin polymerization and cytoskeletal flow at the IS are well understood, the mechanism by which actin flow enhances T cell activation has remained elusive. Recent studies point to the involvement of force-induced receptor activation (3), as well as force-driven formation and centralization of signaling microclusters (1, 2, 4, 5). According to this paradigm, early signaling events drive the robust polymerization of F-actin at the IS, which in turn functions to enhance signal transduction events leading to full T cell activation. In this review, we will focus on the mechanisms through which cytoskeletal dynamics in T cells and APCs serve to control mechanosensitive signaling events at the IS and consider how cytoskeletal function can be included in current models of receptor triggering.

## F-Actin Dynamics on the T Cell Side of the IS

**Figure 1:**
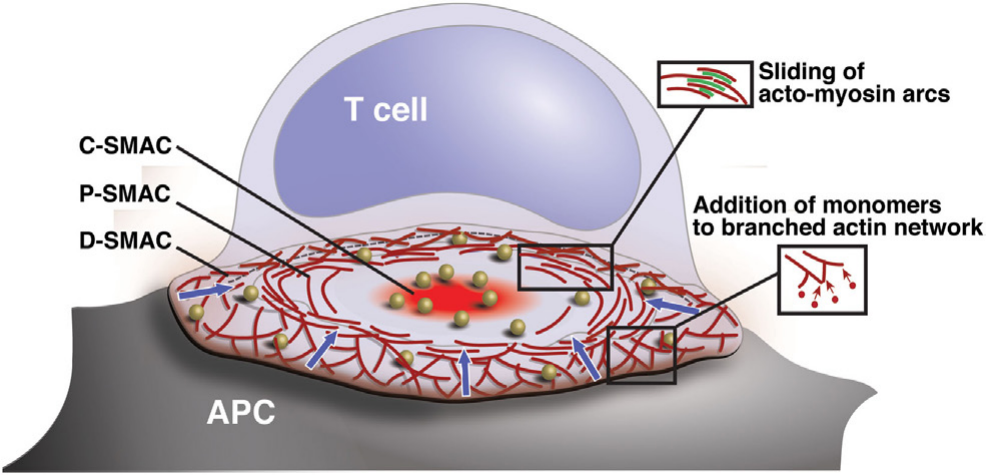
**Organization and actin dynamics within the IS**. Diagram showing the architecture of a radially symmetric “bulls-eye” IS such as that formed between a B cell and an antigen-specific mature T cell. Based on molecular segregation, the IS can be divided into three regions: (1) a peripheral actin-rich region termed the distal supramolecular activation cluster (D-SMAC), (2) a deeper region rich in LFA-1 and actomyosin arcs termed the peripheral supramolecular activation cluster (pSMAC), and (3) a central region rich in PKCθ and other signaling molecules termed the central supramolecular activation cluster (cSMAC). Signaling microclusters containing TCR and other signaling molecules (gold balls) form and begin to signal in the IS periphery and are transported by the cytoskeleton toward the cSMAC region, where signal extinction takes place. Microcluster movement is coupled to centripetal flow of the actin network (blue arrows). Actin flow is driven primarily by addition of actin monomers to the barbed ends of branched actin filaments, which lie just under the plasma membrane. This generates a pushing force that drives the network inward. In addition, myosin-driven sliding of actin filaments causes contraction of the network. This provides a pulling force that stabilizes the network and maintains radial symmetry.

During stimulation by an APC, T cells exhibit robust actin polymerization in the periphery of the contact area, centripetal (retrograde) flow, and eventual disassembly of F-actin filaments near the center of the contact (2, 4) (Figure 1). Consistent with this, actin filaments are generally shorter, more branched, and more dynamic in the periphery of the IS, where nucleation of new actin filaments and polymerization of monomers onto the growing ends of existing filaments are occurring (6). Centripetal flow of the actomyosin network is primarily driven by the polymerization of F-actin, which continuously pushes on the plasma membrane (2, 4). This process is accompanied by the contractile activity of non-muscle myosin IIA, which organizes actin filaments into arcs within the lamellar region. This process stabilizes the network and maintains radial symmetry. Under conditions where F-actin depolymerization is blocked, myosin activity results in network constriction. Simultaneous inhibition of F-actin polymerization, F-actin depolymerization, and myosin contractility results in complete inhibition of lamellipodial actin flow (2, 4). Recently, it has become evident that there are actually two pools of dynamic actin filaments at the IS. In addition to the prominent lamellipodial pool, actin polymerization also takes place in smaller actin foci, structures that are closely associated with newly formed TCR microclusters (7). These foci are likely equivalent to the podosome- or invadopod-like protrusions (ILPs) first visualized in T cells interacting with endothelia, and later also found at the T cell/APC interface (8, 9). Although it has not been directly demonstrated, it seems likely that the conditions shown to arrest lamellipodial actin flow also arrest dynamics of these TCR-associated actin foci.

### Actin-Regulatory Pathways Downstream of the TCR

**Figure 2:**
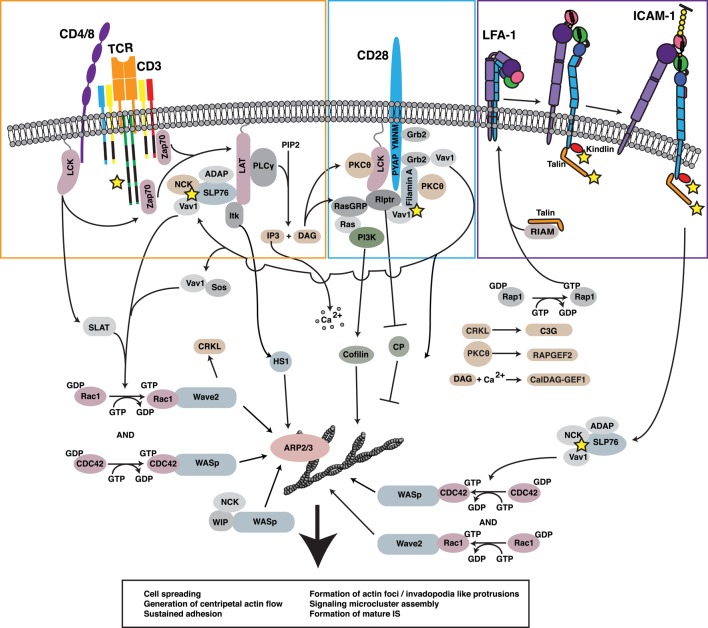
**Regulation of the F-actin network at the IS**. Ligation of multiple receptors, including the TCR, the costimulatory molecule CD28, and the adhesion molecule LFA-1, results in the induction of robust actin polymerization at the IS. The pathways that mediate F-actin polymerization are highly interdependent. For example, the TCR-dependent activation of Lck is involved in initiating CD28-mediated signaling. Moreover, both TCR-induced activation and CD28-induced recruitment of PKCθ contribute to LFA-1 activation and downstream signaling. Vav1, a GEF for the critical actin regulators Rac1 and CDC42 and their respective effectors WAVE2 and WASp, is triggered in a co-operative fashion downstream of each of these key surface receptors. In addition, signaling events downstream of CD28 lead to inhibition of capping protein and activation of cofilin, events that allow growth and remodeling of the branched actin network. Together with HS1, WAVE2 and WASp activate ARP2/3 complex-dependent growth of branched actin filaments, forming lamellipodial protrusions and invadopodium-like protrusions, respectively. Collectively, these events lead to cell spreading, retrograde actin flow, and formation of the mature IS, along with assembly and centripetal flow of TCR-associated signaling microclusters. In addition to triggering actin nucleation, TCR, CD28, and LFA-1 all associate with the F-actin network (proteins known to interact with F-actin are denoted by yellow stars). In many cases, these interactions with the F-actin network serve to drive additional signaling events *via* actin-dependent feedback loops.

Within the T cell, multiple signaling pathways, including those downstream of the TCR, CD28, and the integrin lymphocyte function-associated antigen 1 (LFA-1), lead to the activation of actin-regulatory proteins (Figure 2). The relevant signaling pathways downstream of the TCR have been reviewed extensively (10–12) and will only be briefly discussed here. Following TCR engagement, several protein tyrosine kinases, including Lck and ZAP-70, are activated, leading to the phosphorylation of multiple effectors. One key effector is the scaffold protein linker for activation of T cells (LAT). LAT phosphorylation recruits SLP-76 to the IS, and with it the Rho-family GTPase exchange factor (GEF) Vav1, the adapter Nck, and the IL-2-inducible T cell kinase (Itk). Activation of Vav1 and other GEFs, such as PIX and SLAT (13–15), leads to GTP loading and activation of the small GTPases CDC42 and Rac1. These GTPases, in turn, recruit and activate the actin nucleation promoting factors WASp and WAVE2, which work in concert with the related protein HS1 to orchestrate Arp2/3 complex-dependent polymerization of branched actin filaments (16–18).

Interestingly, WASp, WAVE2, and HS1 play distinct roles in organizing lamellipodial actin and actin foci. WAVE2 localizes strongly to lamellipodial protrusions and is essential for their generation (17, 19), whereas WASp is largely dispensable for generation of these structures (20). Instead, WASp localizes to and is essential for the formation of TCR-associated actin foci (7), further extending the similarity between these structures and podosomes in other hematopoietic cells (21, 22). The role of WAVE2 in generating actin foci cannot be meaningfully tested because WAVE2-deficient T cells do not spread in response to TCR engagement, but WAVE2 is absent from these structures (7). HS1 can be found in both lamellipodia and actin foci, and in its absence, both sets of structures are disordered (7, 16). Thus, it appears that WAVE2 organizes lamellipodia that result in T cell spreading on the APC, WASp organizes TCR-associated foci that protrude into the APC, and HS1 augments and organizes both sets of actin-rich structures.

### Integrin-Mediated Organization of the T Cell F-Actin Network

Another effect of TCR signaling is to induce conformational changes in LFA-1, an integrin that mediates IS formation and firm adhesion (23). LFA-1 engagement initiates a signaling cascade that parallels and intersects with the TCR-triggered cascade. This process has been termed “outside-in” signaling to distinguish it from “inside out signaling” events that trigger initial integrin activation downstream of TCR or chemokine receptor engagement. Molecules activated downstream of LFA-1 engagement include FAK, ERK1/2, JNK, and PLCγ1 (24–26). LFA-1 regulates F-actin through the ADAP-mediated activation of SLP-76 (27–29). This results in F-actin polymerization, likely through the Vav-mediated activation of Rac1, CDC42, WASp, and WAVE (Figure 2) (30–32). Recruitment of the Arp2/3 complex to the site of integrin engagement is enhanced by interactions of the complex with the talin-binding protein vinculin (32–34). As discussed later, integrin activation and vinculin binding to talin are dependent on the interaction of talin with the F-actin network and on ongoing F-actin flow. This suggests a robust feed-forward loop whereby integrin activation is dependent on F-actin-generated forces and results in increased activation of F-actin nucleating factors and polymerization at the IS.

Although integrin engagement can induce actin polymerization, it can also modulate F-actin flow rates. Engagement of VLA-4, a β1 integrin expressed on activated T cells, by immobilized VCAM-1 greatly decreases the centripetal flow of F-actin at the IS (35). This likely occurs through the interaction of multiple actin-binding proteins with the β chain of VLA-4, thus linking the ligand-immobilized integrin to the F-actin network and retarding network flow (35, 36). So, while integrins are capable of nucleating F-actin polymerization, the overall effect on the F-actin network will depend on the strength of the outside-in signal, the interaction between the integrin cytoplasmic domain and the actin network, the viscoelastic properties of the network itself, and the mobility of the integrin ligand (since only immobilized ligand could oppose forces on the integrin tail).

### Costimulatory Signals Leading to F-Actin Remodeling

Coligation of the costimulatory molecule CD28 with the TCR leads to robust IL-2 production, activation, and expansion of naive T cells (37). The classical pathways involved with CD28 costimulation have been extensively reviewed (38–41). As part of this process, CD28 signaling regulates F-actin dynamics. CD28 can interact with F-actin through binding to filamin A (Figure 2). By binding to the adapter protein Grb-2, CD28 also promotes the formation of Vav 1/SLP-76 complexes and initiates downstream signaling (42–44). In cells in which Csk, a negative regulator of Lck, has been inhibited, CD28 binding to CD80/86 can mediate robust F-actin polymerization (45). CD28-dependent F-actin polymerization occurs through Vav-mediated activation of CDC42 and is enough to initiate cell spreading, though the appearance of the F-actin network is not as symmetrical as with TCR stimulation (46). CD28 costimulation has also been shown to induce the dephosphorylation and activation of the actin-severing protein cofilin (47). Somewhat counter-intuitively, actin severing by cofilin can increase rates of actin polymerization by providing actin monomer and freeing otherwise capped barbed ends (48). The overall effect of increasing both F-actin severing and polymerization is to create a highly branched F-actin network, a process that can strengthen lamellipodial protrusions and contribute to F-actin flow. Another molecule that is likely to participate in CD28-dependent actin responses is the lymphoid cell-specific actin-uncapping protein, Rltpr. As detailed in Section “Regulation of CD28 Signaling by the F-Actin Network,” Rltpr interacts with CD28 and plays an essential role in costimulatory signaling (49). It remains to be determined if Rltpr functions to remove capping protein from barbed ends of actin filaments at the IS, but if so, this will also be important for F-actin remodeling.

In addition to CD28, it is likely that many other costimulatory proteins also modulate the T cell actin response. One protein known to interact extensively with F-actin is CD2. CD2 is expressed on the surface of NK cells and T cells, and it can mediate cell adhesion and induce signaling events that promote T cell activation (50, 51). Through the cytoplasmic adaptor molecule CD2AP/CMS, CD2 engagement can recruit and activate capping protein, cortactin and WASp, facilitating the formation of a short, branched actin network (52–56).

## The F-Actin Cytoskeleton and the Control of Molecular Activation at the T Cell–IS

As detailed above, multiple signaling cascades converge to initiate and control F-actin flow at the IS. Conversely, however, F-actin dynamics are critical for proper signal transduction. Thus, a positive feedback loop exists whereby initial signaling events induce F-actin restructuring, which in turn reinforces and sustains signaling. In the following sections, we will describe the mechanisms by which the F-actin network can control or mediate signaling events on the T cell side of the IS.

### Maintaining Quiescence in Resting Cells

The maintenance of T cells in a quiescent state in the absence of cognate antigen is critical for the prevention of autoimmunity and the proper regulation of the immune response as a whole. To maintain quiescence, T cells make use of several mechanisms. Based on work in B cells, one likely mechanism involves segregation of signaling molecules into separate cell surface compartments. As has been shown for the B cell receptor (BCR) (57), the T cell actin cytoskeleton may limit baseline signaling by preventing clustering of the TCR or downstream signaling intermediates. In fact, one way that antigen experienced cells maintain increased sensitivity to antigen is through the oligomerization and clustering of the TCR, suggesting that this process is, in fact, regulated (58). Additionally, it has been reported that large clusters of TCR and LAT are maintained separately in resting cells, and only overlap upon activation (59). Although LAT clusters are maintained by the actin cytoskeleton, it remains possible that actin also separates LAT and TCR clusters in resting T cells (60). Reorganization of the actin network following stimulation could then permit or drive cluster growth and molecular interactions. In B cells, actin-binding proteins of the ezrin, radixin, moesin (ERM) family limit BCR cluster formation, preventing aberrant signaling through the maintenance of diffusional barriers (57). BCR signaling transiently deactivates ERM proteins, allowing for increased BCR diffusion and cluster formation. This cycle is required for antigen capture, as both constitutively active and dominant negative ERM proteins interfere with this process (61). This shows that while ERM-mediated diffusional barriers may aid in maintaining a quiescent state, these barriers also undergo a dynamic cycle of activation and deactivation. A similar process may be occurring in T cells, since TCR stimulation also causes ERM protein dephosphorylation and cytoskeletal relaxation (62).

### T Cell Migration, Initial Antigen Scanning, and the Conversion to a Stable IS

In many experimental systems, T cells are introduced to stimulatory surfaces from suspension, such that initial TCR-induced actin polymerization is required for cell spreading and synapse formation. *In vivo*, however, initial contact between T cells and APCs occurs within the context of T cell migration. T cell migration requires actin-mediated protrusion of the leading edge and myosin-mediated contraction of the trailing uropod (63–65). Initial T cell scanning is characterized by short-lived T cell/APC interactions. During these interactions, T cells form mobile synapses known as kinapses, which exhibit protein segregation patterns analogous to those seen in mature synapses, but are not radially symmetrical (66, 67). In essence, then, the conversion between kinapse and synapse entails altering the symmetry of the actomyosin network. This appears to be determined, at least in part, by the strength of TCR signaling. In support of this, the balance between PKCθ signaling and WASp activity determines if cells are likely to break or maintain symmetry (68). Although additional details of how T cells maintain this balance are yet to be worked out, it has been proposed that PKCθ fosters symmetry breaking by activating localized myosin contractility (67). In addition, there is evidence that intracellular calcium levels also play an important regulatory role (69–71).

### Coupling of Signaling and Actin-Driven Microcluster Dynamics

Following the formation of a stable, symmetric synapse, microclusters of TCR and downstream signaling components, such as Zap70 and SLP76, form in the periphery of the IS (peripheral supramolecular activation cluster, pSMAC) and undergo transport to the center of the contact zone (central supramolecular activation cluster, cSMAC). Depolymerization of F-actin abolishes the generation of new TCR microclusters, as well as inward movement of existing TCR microclusters (1, 72), but the mechanisms linking the actin cytoskeleton to microcluster formation and movement have yet to be fully worked out. Since microtubules and cytoplasmic dynein have been implicated in microcluster movement toward the cSMAC (73), one could imagine a model in which the actin network functions as a static scaffold for microcluster nucleation, with subsequent microtubule-dependent microcluster transport contingent upon maintenance of this actin scaffold. However, this model has been ruled out; when cells are treated with an inhibitor cocktail that arrests actin dynamics but leaves the network intact, the formation and translocation of SLP76 microclusters are blocked (2). Furthermore, actin flow rates are locally perturbed at TCR microclusters that encounter a barrier to inward transport (74), suggesting direct interactions between the TCR and the actin network. Although it remains unclear exactly how actin dynamics promote continued signaling from individual microclusters, arresting F-actin dynamics interrupts phosphorylation of PLCγ, resulting in a rapid drop in intracellular Ca^2+^ levels (2). Actin foci are likely to be the relevant actin-rich structures in this context, since loss of WASp (or HS1) inhibits PLCγ1 activation and associated Ca^2+^ signaling, while loss of WAVE2 affects Ca^2+^ signaling at the level of CRAC channel coupling, leaving PLCγ1 activation intact (17).

In addition to driving microcluster formation and sustaining signaling, IS-associated F-actin flow sets a molecular countdown for signal termination. Tyrosine phosphorylation of early signaling intermediates typically occurs in microclusters located in the pSMAC (72), whereas the cSMAC is an area of protein dephosphorylation, ubiquitinylation, and internalization to form IS-associated microvesicles (75, 76). Prolonging the time microclusters spend in the cell periphery actually prolongs signaling lifetime (72, 77). For example, recruitment of TCR into the cSMAC is dependent on the ubiquitin-binding protein TSG101, and knockdown of TSG101 inhibits cSMAC formation and increases microcluster lifetime and total phosphotyrosine levels at the IS (76). Thus, while dynamic actin filaments first initiate the formation of active signaling microclusters, they subsequently lead to their deactivation by driving their accumulation at the cSMAC. Interestingly, formation of TCR-enriched microvesicles occurs as a linear function of MHC density (75). Moreover, the amount of active signaling that occurs within the cSMAC varies with peptide dose and agonist strength (78). Thus, signal activation and extinction can be modulated at the level of microcluster dynamics, to tune T cell responses over a broad range of antigenic signals.

### Force Generation and T Cell Activation

During the initial contact between a migrating T cell and an APC, and in the radially symmetric mature synapse, multiple forces are applied to the molecular contacts between the two cells. As T cells migrate on the APC surface, actin polymerization at the leading edge and myosin contractility at the trailing uropod provide this force, while at the mature IS, the retrograde F-actin flow provides a similar force. With this in mind, molecular contacts between TCR and pMHC, integrins and integrin ligands, and costimulatory molecules and their ligands must persist and signal under constant strain. Interestingly, the generation of molecular forces at the IS downstream of pMHC–TCR interactions is directly correlated with the antigenicity of a given pMHC (79), and T cells respond differently depending on the mechanical properties of the stimulatory surfaces they encounter (80, 81). Over the stiffness ranges tested so far, it has been shown that human T cells respond better to substrates of increasing stiffness, and this corresponds to an increased ability to generate force at the IS along with increasing substrate stiffness (81, 82). Additionally, migrating T cells are far more sensitive to antigen when encountered at the leading edge, rather than at the less dynamic uropod, suggesting that the forces at the leading edge prime the TCR to respond to cognate antigen (83, 84). This evidence suggests that mechanical force is integrally involved in T cell activation. If this is the case, then studying the mechanical forces on the TCR and other receptors at the IS and the relevant mechanosensitive signaling pathways becomes critically important in gaining a complete understanding of T cell activation.

### Cytoskeletal Forces and the Initiation and Maintenance of TCR Signaling

Although the molecular interactions between the TCR and pMHC have been extensively characterized, the mechanism by which information on receptor ligation is transmitted across the plasma membrane and transformed into the biochemical signals associated with TCR triggering is unknown and hotly debated. Several challenges unique to the TCR/pMHC interaction must be overcome in order to initiate signaling, and any model proposed to describe TCR triggering must take these into account (85). First, TCR triggering must be extraordinarily sensitive, as there are typically only a few molecules of cognate pMHC on the surface of a given APC. Indeed, TCR triggering and T cell activation can occur in response to a single pMHC complex (86). Second, the TCR must efficiently discriminate between rare agonist and plentiful non-agonist pMHC molecules. Finally, TCR triggering must occur despite a near limitless diversity in the binding of pMHC and TCR. Several models have been proposed to account for these requirements. It is illuminating to consider these models in terms of the potential role of forces generated by F-actin at the IS. It is important to note that many observations that support a role for cytoskeletal force can be explained within the context of multiple models, and it is likely that several mechanisms are working together to initiate TCR triggering.

#### The Kinetic-Segregation Model

The kinetic-segregation model was proposed, in part, to account for the large proportion of Lck that is phosphorylated on the activating tyrosine, Y394, even in the absence of TCR stimulation (87). It is likely that this active Lck is continuously opposed by the action of CD45 and other phosphatases, since pharmacological phosphatase inhibition induces T cell activation in the absence of TCR stimulation (88–90). Additionally, removal of the Lck negative regulator C-src tyrosine kinase (Csk) results in the activation of proximal TCR triggering events (45). It is therefore unsurprising that the balance between tonic signaling and activation of TCR signaling depends on the expression of Csk, inhibitory phosphatases such as CD45, and kinases such as Lck (91). The kinetic-segregation model proposes that close membrane apposition enforced by the TCR/pMHC bond length (~15 nm) is too small to allow colocalization of proteins with large extracellular domains, such as CD45. In the model, the TCR is a passive player in this process, and close membrane apposition is driven entirely by the affinity of TCR for pMHC and the formation of multiple bonds leading to stochastic size-based sorting and exclusion of large molecules from pMHC/TCR rich areas. Exclusion of CD45 then allows the system to be dominated by the constantly active Lck, and TCR triggering ensues (92). Indeed, *in vitro* modeling of the TCR signaling network on reconstituted liposomes has shown that clustering of CD3ζ and Lck is enough to overcome even high concentrations of CD45 and induce signaling (93), and large ectodomain proteins have been shown to enhance clustering of smaller proteins and their ligands in live cells (94). Signaling can continue following dissociation of TCR/pMHC as phosphorylated ITAMs can be protected by interaction with their specific binding partners (93).

The strongest evidence for the kinetic-segregation model is based on observations that truncation of the CD45 ectodomain (creating a shorter molecule) impairs TCR-mediated signaling, and that full function can be restored by simply adding any large ectodomain to truncated CD45 (95, 96). Additionally, the size of the ectodomain influences segregation of CD45 and TCR into separate protein islands, with larger ectodomains resulting in greater separation. Moreover, extending the length of the extracellular domain of pMHC by the addition of various length tethers results in poor T cell activation corresponding to greater distances between the APC and T cell, and poor exclusion of CD45 from both the interfaces and from CD3 clusters (97, 98). Though elongated pMHC does not affect TCR or coreceptor binding or TCR clustering, it remains possible that elongation of pMHC affects the force transduced to the TCR, an idea that will be considered below.

Despite the evidence in favor of the kinetic-segregation model, several key problems have arisen in the literature. First, some authors have found that small ectodomains can result in CD45 exclusion from TCR and CD2 microclusters, as well as the total IS interface, suggesting that ectodomain size may not be the only contributing factor in this process (91, 99). Additionally, truncation of the intracellular domain of CD43 results in poor exclusion from the IS, suggesting that segregation based on size is not enough to determine molecular sorting at the IS for all large molecules (100). In these instances, molecular crowding and active transport may also be involved. Second, in NK cells, where similar molecular sorting events separate inhibitory and activating receptors based on ectodomain size (101), it has been found that segregation depends largely on the surface expression level of the small ectodomain protein and its receptor; more expression (and more receptor–ligand engagement) leading to greater segregation (102). This makes sense in that, in order to exclude large ectodomain proteins, the combined bond strength between shorter molecules and their ligands must be strong enough to deform the local plasma membrane and bring cells into close proximity, overcoming resistance posed by the entire glycocalyx. Given the generally low affinity of the TCR for pMHC, multiple interactions would be needed to provide this force. This idea is troublesome given recent evidence that only one pMHC can induce the formation of a microcluster containing hundreds of TCRs, presumably excluding CD45, on a responding T cell (86). Finally, it has been shown that TCR microclusters that exclude CD45 can form in the absence of agonist pMHC, and even in the complete absence of MHC on artificial surfaces coated with ICAM-1 (103). This observation necessitates a different mechanism besides stochastic exclusion of large molecules following TCR/pMHC bond formation to explain any size-based exclusion of CD45 from TCR microclusters.

**Figure 3:**
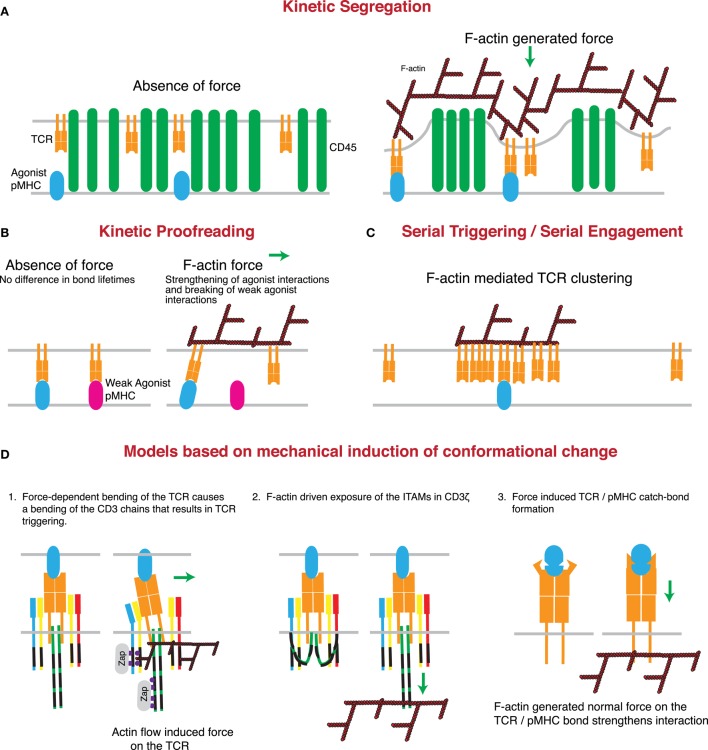
**Mechanisms through which actin-dependent forces can contribute to proposed models controlling TCR triggering**. **(A)** The kinetic-segregation model of TCR signaling is dependent on the separation of molecules with small extracellular regions, such as the TCR, from those with large extracellular regions, such as the phosphatase CD45. Actin-dependent protrusions would serve to bring the T cell and APC plasma membranes into close proximity, thereby driving molecular segregation. This should occur even in the presence of low numbers of cognate pMHC on the APC surface. **(B)** The kinetic proofreading model proposes that TCR triggering is based on longer bond lifetimes for strong agonists than weak agonists. The force-dependent catch-bond behavior of the TCR with strong, but not weak, agonist pMHC complexes can enhance bond lifetime for strong agonists, while serving to rupture the slip-bonds formed by TCRs engaging weak or non-agonist pMHC. **(C)** The serial triggering/serial engagement molecule could benefit from the presence of multiple F-actin interactions with the TCR. Though it may not be force dependent, the association of the TCR with the F-actin network could lead to clustering of the TCR on the plasma membrane, allowing for rapid successive unbinding and rebinding, and serial triggering of multiple TCRs by a single pMHC complex. **(D)** Several conformational changes that have been described for the TCR may be induced or enhanced by the application of force by the T cell actin cytoskeleton. The first posits a mechanical lever-type action of the TCR under the application of a tangential force. According to this model, bending of the stiff CD3 chains propagates to the intracellular domain and results in signal initiation. The second model suggests that actin associations with the CD3 complex help to pull the CD3 chains away from the inner leaflet of the plasma membrane, thus exposing the ITAMs for phosphorylation and binding of essential regulators such as the kinase ZAP70. The third model is based on catch-bond molecular interactions between TCR and cognate pMHC complexes. According to this variant of the kinetic proofreading model, cytoskeletal force causes a conformational change in the TCR that results in stronger pMHC binding and prolonged or enhanced signaling.

The polymerization of the F-actin network and forces generated by the network may be enough to overcome these limitations. During kinapse formation, the T cell actin network is undergoing dynamic regulation through a combination of chemokine receptor, costimulatory molecule, and integrin-mediated signaling. This reorganization of the F-actin network may be enough to push the T cell and APC membranes close together, overcoming the charge repulsion presented by the glycocalyx (104). This force could initiate CD45 exclusion from the TCR in areas of close membrane apposition, even in the absence of TCR/pMHC interactions. Following TCR engagement, forces generated by F-actin polymerization could work in concert with the close membrane apposition enforced by TCR/pMHC interactions to propagate this process, further separating CD45 and other large ectodomain proteins from TCR microclusters, and ultimately excluding them from the mature IS. In line with this idea, it has recently been found that T cells can produce invadosome-like protrusions into the membrane of an APC. These protrusions can form in the absence of antigen (though their frequency and longevity are increased in the presence of antigen) and induce extremely close membrane apposition, overcoming charge repulsions mediated by the glycocalyx (8). Critically, these protrusions (which presumably correspond to the WASp-dependent actin foci described above) are completely dependent on the F-actin network, occur in multiple T cell/APC models, and precede the onset of early TCR triggering. This phenomenon likely explains why disruption of the F-actin network prevents the formation of new TCR microclusters, even in the continued contact between the T cell and an artificial APC (1), since receptor clustering would depend on proximity to pMHC, and CD45 exclusion. Based on this evidence, the kinetic-segregation model can be modified to account for the contribution of F-actin-generated force in initiating close membrane apposition, particularly in the presence of low pMHC concentration, thus contributing to CD45 exclusion form sites of TCR–pMHC binding (Figure 3A).

#### The Kinetic Proofreading Model

The kinetic proofreading model of TCR triggering, initially proposed by McKeithan, posits that TCR triggering requires individual bond lifetimes above a certain threshold duration, and longer than the dissociation time (105). Furthermore, if unbinding occurs prior to this threshold being reached then no signaling occurs and the TCR resets itself. This model was later refined to allow for retention of TCR signaling intermediates, so that rebinding of pMHC to the same TCR would continue from where the previous interaction left off (106). This fits well with the observations that fast pMHC on rates can overcome low dwell times/high off rates and lead to high apparent affinities and TCR triggering. That is, if a pMHC rebinds prior to diffusing away from the TCR, it could induce TCR triggering by reaching the threshold even when any given interaction is particularly short (107, 108). In fact, in 2D experimental paradigms, *k*_on_ has been shown to be one of the best predictors of pMHC agonist strength (109, 110).

Force produced by the F-actin network may play an interesting role in the kinetic proofreading model. It has recently been shown that the TCR can engage in catch-bond molecular interactions, in which applied force prolongs the interaction time with cognate pMHC (111). In that study, Liu et al. show that in the absence of force on the TCR/pMHC bond, there is an inverse relationship between the average lifetime of the bond and pMHC potency. However, following the application of 10 pN of exogenous force to the bond, agonist pMHC bond lifetimes increase, behaving like catch-bond molecular interactions, while antagonist bond lifetimes decrease, behaving like more traditional slip-bond type molecular interactions. This leads to a 57-fold increase in the ratio of bond lifetimes between strong agonist and strong antagonist peptides following the application of force. Additionally, catch-bond behavior correlated strongly with the strength of the agonist (as measured by T cell stimulatory capacity) such that the strongest agonist pMHC had the largest increase in bond lifetime following the application of force and required the greatest force to induce the catch-bond behavior. Interestingly, it has been shown that at the IS, the actin cytoskeleton acts to decrease the half-life of some TCR/pMHC bonds (109). Thus, internally generated force provided by the F-actin network could function similarly to the external force applied in the study by Liu et al. In terms of the kinetic proofreading model, force would thus allow for increased specificity and greater bond lifetimes for agonist vs. antagonist pMHC, enhancing sensitivity and diminishing noise during TCR signal acquisition (Figure 3B).

#### The Serial Triggering/Serial Engagement Model

The serial engagement model was proposed as a way of accounting for the high specificity of the TCR, despite low 3D affinities and high off rates, and low numbers of agonist pMHC on the APC surface. In this model, pMHC serially engages with multiple TCRs, triggering each one individually before moving onto another, and thereby taking advantage of the high off rate to trigger multiple receptors (112). Later studies have confirmed that a single pMHC is capable of recruiting hundreds of TCRs into a complex, initiating T cell activation and cytokine production (86). It has previously been proposed that actin-induced apposition of the T cell and APC membranes would bring the TCR into close proximity to pMHC complexes, accommodating the fast on-rates characteristic of agonist pMHC (113). It is possible that in addition to facilitating single pMHC/TCR interactions, the actin cytoskeleton also serves to bring additional TCRs into the immediate vicinity of ligated TCR/pMHC pairs. This would increase activation efficiency by reducing the time it would take for pMHC to encounter another TCR. The actin cytoskeleton is critical for the formation of TCR and signaling microclusters following simultaneous engagement of multiple TCR molecules (1, 2). It is therefore possible that TCR clustering induced by a single pMHC is also induced or stabilized by the F-actin network, thereby leading to enhanced TCR triggering (Figure 3C). In other systems, direct tethering of transmembrane proteins to cortical actin induces nanoclustering (114). The TCR associates with the F-actin network through both ITAM-dependent and -independent mechanisms (115–117). Although the ITAM-dependent mechanism requires phosphorylation by Lck and is therefore likely to take place after initial TCR triggering, the ITAM-independent mechanism is mediated by two RRR sequences in the CD3ζ chain and causes the constitutive association with F-actin. This association is essential for clustering of the TCR, IS formation, and T cell activation following TCR engagement. Thus, the constitutive and inducible interactions between TCR and F-actin could produce localized increases in TCR concentration, thereby facilitating serial engagement.

### Conformational Change and the Mechanical Induction of TCR Triggering

Recently, the idea that conformational change and mechanosensing may play a critical role in TCR triggering has gained significant traction (3, 12, 118). Structural studies demonstrate the existence of several conformational changes that can occur upon pMHC binding (119–122). In many cases, however, the documented changes in TCR structure did not propagate away from the pMHC-binding site. Conformational changes in the constant domain, away from the antigen-binding site, were subtle, and it remains unclear if these represent conserved changes found in all triggering interactions. Furthermore, it is unclear how such small changes can propagate to the intracellular portion of the CD3 chains. This brings us to the one key problem faced by models proposing conformational changes initiated in the TCR by pMHC binding alone. Specifically, any conformational change must be present in all TCR/agonist pMHC interactions and absent from TCR/non-agonist pMHC interactions. Given the near limitless variation in the TCR- and pMHC-binding sites, it is hard to imagine that all productive interactions occur with a given binding geometry necessary to initiate the same structural changes. In support of this, activating antibodies can perform their function in the absence of any overt structural change to the TCR structure in solution (123). Further complicating the matter, multiple groups have observed that soluble monomeric pMHC is poorly suited to activating T cells, even at extremely high concentrations (124–128), despite TCR/pMHC half-lives otherwise associated with TCR triggering in a 2D environment (109). Finally, as mentioned earlier, simple elongation of the pMHC reduces TCR triggering despite maintaining efficient binding to the TCR, again suggesting that binding-induced conformational change is unlikely to represent a complete TCR triggering mechanism. Interestingly, by incorporating slight modifications to the conformational change model that take into account the cell biology of TCR/pMHC interactions at the IS, one can overcome all of these problems (Figure 3D).

Within the IS, the TCR is dynamically associated with the F-actin network through multiple direct and indirect interactions (Figure 2) (6, 74, 116, 117, 129, 130). These interactions allow F-actin-generated force to be applied to the TCR *via* the actin–TCR linkage. Any resistance to this force provided by surface-bound pMHC could then be converted into a conserved conformational change in the TCR. One key result of refocusing the driver of conformational change from molecular interactions occurring at the site of pMHC engagement to mechanical force applied on the TCR is that these changes in protein structure can occur regardless of the specific molecular contacts occurring between the TCR and pMHC. As long as the interaction is of sufficient affinity to stay bound in the presence of force, productive TCR triggering will ensue, thus overcoming the challenge created by the diversity in pMHC/TCR interactions. Additionally, this mechanism does not require that conformational changes occur within the ectodomains of the TCR subunits; it works equally well for changes in ITAM-containing intracellular domains (131).

Several lines of evidence support the existence of a mechanotransduction-based mechanism for TCR triggering. As mentioned earlier, observations that soluble monomeric pMHC cannot initiate efficient TCR triggering pose a particular problem for the conformational change model (124). Interestingly, surface anchoring of monomeric pMHC overcomes this limitation as low numbers of surface-bound monomeric pMHC can initiate TCR triggering (86, 132, 133). In part, this sensitivity to pMHC and continued signaling is dependent on an intact cytoskeleton, as addition of actin depolymerizing agents causes rapid loss of calcium flux without loss of IS formation (1, 132, 134). This effect of actin inhibition is specific to 2D stimulatory settings, since actin depolymerization when the TCR is cross-linked in solution leads to prolonged calcium responses (135). Moreover, inhibition of cytoskeletal dynamics under conditions that retain the actin scaffold also results in a drop in intracellular calcium (2), showing that dynamic actin plays an active role. A likely explanation for these observations is that in the 2D setting, as opposed to monomer in solution, the dynamic actin filaments can generate force on the pMHC/TCR bond, thereby initiating signaling. Supporting this idea, tangential force applied on the TCR through non-activating antibodies can result in initiation of calcium flux. Strong stimulatory anti-CD3ϵ antibodies may mimic this force by binding to the side of the complex in a way that induces a bending of the CD3 molecule, in contrast to non-activating antibodies that bind more perpendicularly at the membrane-distal portion of the CD3 complex (136). These data support a model in which force on the TCR/pMHC complex applied tangentially, and not normally, makes the TCR act as a lever, bending and activating the associated CD3 complexes (Figure 3D, model 1) (137). Critically, the actin flow at the IS is radially symmetric and directed toward the center of the IS. Force vector measurements at the IS show that this actin flow results in a similarly directed force applied to the substratum through the TCR (138, 139). This force is consistent with the F-actin-driven centralization of TCR microclusters and would apply a tangential force on the TCR/pMHC bond.

Interestingly, an external normal force can also initiate TCR triggering, though whether normal and tangential forces act by the same or different conformational triggering mechanisms is unknown (140). Critically, it was shown that simple contact between the TCR and pMHC probe was insufficient to induce signaling. Instead, continuous force was required to maintain calcium flux; signaling stopped and resumed with the cessation and reapplication, respectively, of external force. Consistent with this, the loss of TCR triggering that occurs when the extracellular domain of the pMHC is artificially elongated, usually used as evidence for the kinetic-segregation model, can be overcome through the application of tangential or normal force to the TCR/pMHC bond. This finding is important in that it suggests that prior findings, interpreted as support for the kinetic-segregation model, can be reevaluated to fit into a coherent theory of TCR triggering based on the application of force on the TCR/pMHC bond. Although these findings strongly suggest the existence of conformational changes induced through the application of normal and/or tangential force, the structural nature of these changes with each type of force is still unclear. This is complicated by the fact that conformational changes under strain are particularly difficult to study, as they are not likely to exist with pMHC binding to purified, soluble, TCR components.

How exactly force is applied to the TCR is an important question. As already mentioned, the TCR can interact both directly and indirectly with the actin cytoskeleton. The direct association of CD3ζ is mediated by two stretches of basic amino acids, and mutation of these residues results in decreased synapse formation and T cell activation (116). Interestingly, these same amino acid stretches also mediate binding of the CD3ζ to the negatively charged inner leaflet of the plasma membrane, limit the phosphorylation of ITAMs (141, 142), and are required for synaptic recruitment of CD3 (143). The dual role of the basic stretch suggests a possible competition of binding for the basic residues in the CD3ζ cytoplasmic domain, with binding to the inner leaflet acting as a negative regulator for activation, and binding to the actin cytoskeleton acting as a positive regulator. It also raises the possibility that following ligand binding, force exerted by the actin cytoskeleton on CD3ζ may physically disrupt the association of CD3 chains with the plasma membrane, helping to expose the ITAMs for subsequent phosphorylation (Figure 3D, model 2). Similar binding of the CD3ϵ chain to the plasma membrane also restricts phosphorylation of ITAMs within the ϵ chain by Lck (144). Though no direct CD3ϵ/F-actin interaction has been discovered, it is known that CD3ϵ can bind directly to Nck following TCR engagement prior to detectable phosphorylation of ITAMs, and the Nck-binding site is exposed following TCR engagement, and independently of TCR signal initiation (131, 145). This interaction is critical for the initiation of TCR triggering at very early steps, since mutating the residues involved in Nck binding or blocking the interaction with cell permeant peptides results in greatly diminished phosphorylation of CD3ζ, CD3ϵ, and Zap70, reduced recruitment of CD3 to the synapse, and inhibition of proliferation and effector function (146, 147). Nck is linked to actin polymerization through recruitment and binding of N-WASp and WASp (148, 149). Since an N-WASp-mediated linkage between actin and p130 Cas has been proposed to cause the force-dependent activation of p130 Cas (150), it is likely that the connection of CD3ϵ to the F-actin network through Nck can transduce a similar force. Therefore, CD3ϵ ITAM phosphorylation could be regulated by actin-generated force in a way similar to the one proposed for CD3ζ.

An alternate model involving normal force stems from recent work by Liu et al. demonstrating that TCR/pMHC interactions show catch-bond behavior (111). Since many of the theoretical mechanisms for catch-bond formation require an accompanying conformational change (151), this study provides strong circumstantial evidence for the existence of an as-yet undefined conformational change at the site of TCR/pMHC interaction. In the study by Liu et al., a tensile normal force was applied by retraction of an extracellular probe bearing pMHC, though it has been theorized that a similar normal force can be generated internally through the action of the F-actin network (118). In this case, the F-actin flow at the IS would pull on the TCR, inducing a conformational change in the ectodomain that would strengthen the interaction with bound pMHC complexes (Figure 3D, model 3). Even if this conformational change does not initiate signaling, it could enhance the probability of TCR triggering, as in the kinetic proofreading model.

### Regulation of Integrin Function by Cytoskeletal Forces

**Figure 4:**
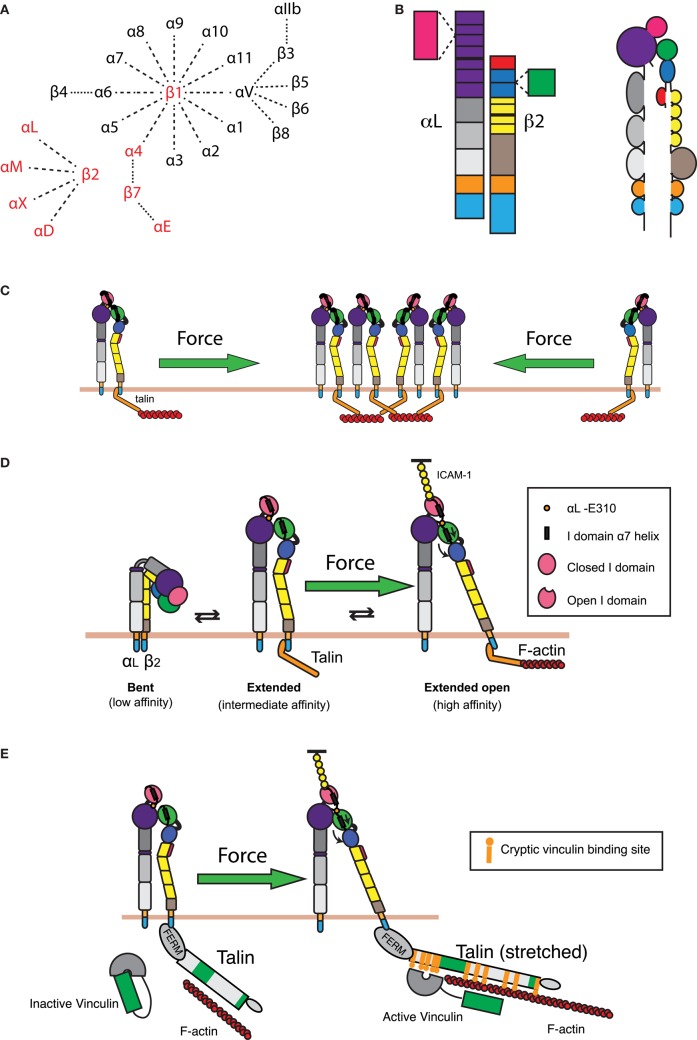
**Integrin regulation by cytoskeletal forces**. **(A)** Known α and β integrin chains and pairings. Integrin pairs expressed in leukocytes are depicted in red. **(B)** Domain structure of the integrin LFA-1. The α chain consists of an intracellular tail (cyan), a transmembrane domain (orange), two calf and one thigh domains (gray), and a β-propeller domain (purple) with an inserted ligand-binding I domain (pink). The β chain consists of an intracellular tail (cyan), a transmembrane region (orange), a β tail domain (brown), four EGF repeats (yellow), a hybrid domain (blue) with an inserted βI domain (green), and a PSI domain (red). **(C)** Retrograde actin flow drives LFA-1 into the IS from the cell periphery. This increases local concentrations of LFA-1, thereby increasing the valency of the interaction and strengthening cell–cell adhesion. **(D)** LFA-1 can exist in roughly three conformations: a bent, low affinity conformation; an extended intermediate affinity conformation; and an extended conformation, where the hybrid domain on the β chain is swung outward, allowing for downward movement of the α7 helix in the βI domain. This downward movement allows the βI domain to bind an internal ligand in the αI domain, causing downward movement of the αI domain α7 helix and opening of the ligand-binding site. These changes generate a high affinity, extended-open conformation. Maintenance of this conformation at the IS is dependent on ongoing actin flow, presumably because connection of the β chain intracellular domain to the dynamic F-actin network is enough to drive swing-out of the hybrid domain. The resulting force-dependent increase in affinity would promote and augment changes induced by ligand binding. **(E)** In addition to regulating LFA-1 affinity for ligand, applied force can also strengthen the connection of LFA-1 to the underlying actin cytoskeleton. Talin, a key protein that links integrins to the actin network, can stretch upon the application of force. This stretching reveals up to 11 cryptic vinculin-binding sites. Vinculin, itself an actin-binding protein, then binds to the exposed sites and reinforces linkage to the F-actin network.

In T cell/APC contacts, integrins are primarily responsible for the adhesive interactions that maintain cell–cell contact (152, 153). Each integrin consists of an α and a β subunit, paired as shown in Figure 4A. In T cells, integrins are required for firm adhesion to endothelium during diapedesis and for formation of stable T cell/APC interactions, resulting in T cell activation or effector function. As such, integrins must function in a variety of extracellular environments, even under the extraordinary strain placed on the integrin–ligand bonds by the shear flow in blood vessels. Furthermore, integrin activation must be tightly regulated to prevent improper lymphocyte function. In general, integrins are regulated at two distinct levels: affinity (the strength of interaction between each individual integrin molecule and its ligand) and valency (integrin density at the cell–cell interface). Both valency and affinity contribute to adhesion (154). Therefore, the overall strength of interaction, or avidity, is a product of valency, affinity, and relative contact area (155). In T cells, intracellular signals emanating from chemokine receptors or the TCR have been shown to increase the activation state of integrins on the cell surface. This “inside out” signaling can result in either changes in valency or affinity, and a large body of work has accumulated defining the relevant biochemical pathways (155–157). Recently, new data have emerged demonstrating the regulation of integrin activation through applied forces (158–160). In the following section, the signaling pathways governing integrin activation at the IS will be covered, with a particular focus on the role of cytoskeletal forces in initiating and sustaining changes in integrin valency and affinity.

The αLβ2 (CD11a/CD18) integrin LFA-1 is expressed exclusively in leukocytes and is essential for T-cell trafficking and IS formation. In resting T cells, LFA-1 is maintained in an inactive, bent conformation with very low ligand-binding capacity (Figure 4D). Signaling through TCR and CD28 results in the activation of the small GTPase Rap1 downstream of PLCγ, PKCθ, and CrkL (161–164) (Figure 2). Following Rap1 activation, talin is recruited to the IS through the action of RIAM, which links talin to the membrane targeting CAAX domain of activated Rap1 (165). Recruitment of talin to the IS is required for LFA-1 affinity and valency modulation as well as conjugate formation (166). Binding of the talin head domain to the cytoplasmic domain of the integrin β chain causes alterations to the β transmembrane domain, thereby relieving interactions between the α and β chains that maintain the bent conformation. This process allows unfolding of LFA-1 and the adoption of an intermediate conformation with 10-fold increased affinity for ligand over the bent conformation (167–175). This “switch-blade” like unfolding occurs in the presence of activating antibodies or ligand-mimetic peptides and exposes epitopes that report on integrin activation (176, 177). Importantly, overexpression of the talin head domain is enough to result in extension of the majority of LFA-1 molecules on the cell surface, but it does not fully rescue cell adhesion, suggesting that the actin-binding domain of talin is essential for full LFA-1-mediated cell adhesion (166, 178). In addition to talin, other proteins, including members of the kindlin family of adaptors, are known to bind to the cytoplasmic domains of integrin β chains and link them to the cytoskeleton, further emphasizing that cytoskeletal linkage is essential for proper integrin function (160, 179).

The role of the cytoskeleton in mediating changes in LFA-1 valency is not straightforward. Early studies proposed a mechanism whereby cytoskeletal restraints limited the mobility of LFA-1 in resting cells, thus preventing clustering. Upon activation, cytoskeletal restraints were released, allowing the free diffusion and coalescence of LFA-1, thus increasing valency (180–182). In this model, the increased association of high affinity LFA-1 with the cytoskeleton limits the ability to support firm adhesion (183). In support of this idea, low dose Cytochalasin D increases LFA-1 mobility and clustering and increases cell adhesion to ICAM-1-coated surfaces. These changes do not induce, and function independently of, changes to integrin affinity and conformational change (154, 184). Importantly, later studies demonstrated that the integrin clustering mediated by actin depolymerization only occurs in the presence of ligand, and suggested a trapping mechanism aided by the increased diffusivity of LFA-1 in the absence of the cytoskeleton (184). This would indicate that LFA-1 interaction with the cytoskeleton limits valency and is in contradiction with the finding that talin, the main link between LFA-1 and the cytoskeleton, is required for LFA-1 synaptic accumulation (166). Further complicating the picture is the observation that transport of microclusters containing LFA-1 and ICAM-1 at the IS is dependent on an intact actomyosin network (4, 185). One of the key confounding factors in this literature is that the studies that identified the actin cytoskeleton as a negative regulator of LFA-1 valency were not carried out within the context of an IS. In an IS setting, actin retrograde flow can actively draw LFA-1 into the IS, increasing the valency of LFA-1/ICAM-1 interactions (Figure 4C).

In addition to the regulation of valency, integrin avidity can be regulated at the level of affinity. Changes in integrin affinity are generally associated with conformational changes (158) (Figure 4D). As previously mentioned, “inside out” regulation of integrin extension mediates the transition from the low affinity to the high affinity conformation. Conformational change from the intermediate to the high affinity state results in a further 100-fold increase in affinity for ligand and has been proposed to be mediated by forces generated by the T cell actin cytoskeleton (159, 175). Structural changes associated with integrin activation have been characterized using activating mutations and antibodies (176, 186–188). Typically, integrin activation and ligand binding are associated with a lateral swing-out of the hybrid domain and downward movement of the α7 helix in the βI domain. This induces the high affinity conformation of the βI domain and has been shown to occur through a series of conformational intermediates (189). In αI domain-containing integrins such as LFA-1, the activated βI domain binds an invariant glutamate residue in the C-linker region between the αI- and β-propeller region. This results in downward movement of the αI domain α7 helix and adoption of the extended open, high affinity, αI domain. Importantly, antibodies that stabilize the extended and extended-open conformations greatly increase LFA-1’s affinity for ligand, resulting in a near 1000-fold dynamic affinity range from the bent to the extended-open conformations (175, 190). Furthermore, shortening of the C-linker region to mimic the downward motion exerted by the βI domain results in constitutively active LFA-1 (187). Steered molecular dynamic simulations have demonstrated that conversion between different integrin conformations can occur through the application of physical forces. Pulling on the headpiece or on bound ligand can overcome interactions between the hybrid and β-tail domains that help maintain the bent conformation, resulting in integrin extension. Importantly, forces applied to the headpiece were not sufficient in these simulations to induce the opening of the headpiece or separation of the integrin legs (191). Interestingly, similar simulations have shown that a tensile force applied parallel to the membrane on the β cytoplasmic tail can be propagated along the β chain, resulting in hybrid domain swing-out and affinity modulation (188). Since talin binds to the integrin β cytoplasmic domain, any force applied on LFA-1 through talin’s linkage to the retrograde actin flow at the IS would result in a similar tensile force, and should mediate integrin affinity maturation (Figure 4E). Intriguingly, it is known that high affinity LFA-1 is more tightly bound to the actin cytoskeleton than intermediate or low affinity LFA-1, supporting the idea that linkage to the underlying cytoskeleton is involved in conformational regulation (192). Our recent work has demonstrated that, indeed, the force provided by retrograde actin flow is critical for maintaining LFA-1 in the high affinity conformation, ligand binding, and clustering of LFA-1 at the IS (193). Thus, connection of LFA-1 to the dynamic actin network provides the force required to initiate integrin recruitment to and clustering within the IS, thereby increasing valency, and also provides the force to induce conformational change to the high affinity state (Figure 4, green arrows).

Consistent with the prediction that force can enhance LFA-1 affinity, integrins engage in catch-bond interactions (194–196). As with other adhesion molecules, such as selectins, integrin bond lifetime increases as tensile normal force is applied, until a threshold known as critical force is reached, where bonds are rapidly ruptured (151, 197). Importantly, blocking binding of the αI internal ligand by the open βI domain inhibits catch-bond behavior, suggesting that conformational change initiated by hybrid domain swing-out is required to initiate catch-bond interactions. Furthermore, it has been shown that α5β1 and LFA-1 bond lifetimes are increased following a short, transient, period of high force application. For LFA-1/ICAM-1 interactions, loading and then unloading of applied force stabilizes the integrin/ligand bond, increasing the average bond lifetime from 1.5 to over 35 seconds (198).

So far, we have discussed the mechanosenstive aspects of LFA-1 regulation with a focus on integrin–ligand interactions. Importantly, the connection between the T cell and the APC mediated by the integrin–ligand bond is only as strong as the weakest link in the pathway (199). Whereas catch-bond interactions between integrins and their ligands exhibit increased affinity with the application of force, the links that connect integrins to the cytoskeleton are thought to behave as more conventional slip bonds, where force decreases bond lifetime. Nonetheless, the talin-mediated linkage of LFA-1 to the actin cytoskeleton is regulated by the application of force. Once talin binds to the integrin β tail through its head domain and to F-actin through its rod domain, actin–myosin-mediated force pulls on talin. This causes talin to unfold like an uncoiling spring, thereby exposing up to 11 cryptic vinculin-binding sites (200, 201) (Figure 4E). Binding of vinculin to the talin rod domain then allows vinculin to bind to F-actin and enforces the integrin linkage to the actin cytoskeleton (160, 201–204). Although this process is reversible, such that loss of force leads to diminished vinculin binding, vinculin binding can stabilize the unfolded conformation of talin (205). In T cells, vinculin is recruited to the IS and is required for talin recruitment and conjugate formation, suggesting that the destabilized talin–F-actin bond is not enough to maintain LFA-1 activation (206). Thus, talin–vinculin binding represents another force-dependent step in the pathway leading to integrin activation, and another mechanism through which cell adhesion can be enhanced by F-actin flow (Figure 4E).

Integrin-mediated outside-in signaling has been mentioned earlier as a driver of multiple pathways of T cell activation. Importantly, the conformational changes that mediate LFA-1 affinity maturation are also required for outside-in signal initiation. Blocking LFA-1 affinity maturation leads to decreased IL-2 production and T cell proliferation (207). Likewise, inhibition of the separation of the transmembrane domains through addition of an inter-subunit disulfide bond results in the loss of outside-in induced stress fiber formation and cell spreading in CHO cells (208). Conversely, affinity modulation through the addition of conformational change-inducing antibodies results in the same pattern of outside-in tyrosine phosphorylation as actual ligand binding. This suggests that integrin conformational changes are necessary and sufficient to induce outside-in signaling. Therefore, forces on the integrin–ligand bond that induce and stabilize integrin conformational change are likely to also be required to initiate and sustain outside-in signal transduction.

Given the accumulating evidence that physical force exerted by the actin cytoskeleton drives conformational changes that mediate LFA-1 activation and stabilize this active conformation, we must re-evaluate our understanding of TCR-mediated integrin activation. Under this new paradigm, forces generated by the retrograde flow of the T cell actin cytoskeleton act as a key component of inside-out signaling and are a critical allosteric regulator of integrin activation at the IS.

### Regulation of CD28 Signaling by the F-Actin Network

**Figure 5:**
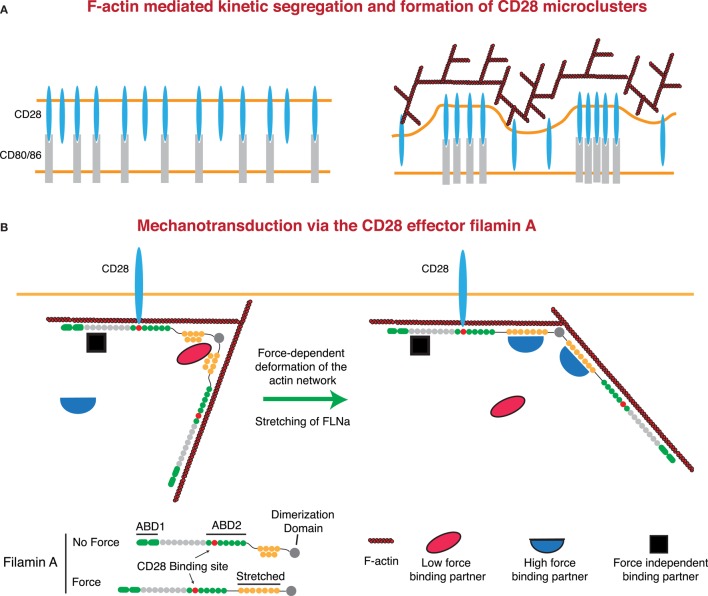
**Regulation of CD28 signaling by applied forces**. **(A)** CD28 clustering at the IS may occur in a signaling-independent manner through the kinetic segregation of bound and unbound molecules. Actin polymerization could contribute to this process by generating protrusions that bring the T cell plasma membrane into close proximity with that of the APC. In the case of a receptor and a surface bound ligand, the free receptor would occupy less extracellular space than the bound receptor–ligand pair, such that bound receptors would be forced to cluster within areas of low membrane proximity. **(B)** Filamin A (FLNa) is a scaffolding protein and an essential CD28 effector that can undergo force-dependent conformational change. Force causes the extension of the second rod domain of filamin A, eliminating binding sites that exist in the relaxed protein, and exposing a different set of binding sites that only exist in the extended protein. Thus, under tension, FLNa can release low force-binding partners and recruit new proteins to the CD28 signaling complex.

As with the TCR and LFA-1, there is strong evidence that F-actin contributes to costimulatory signaling at the IS. Here, we will focus on CD28, although signaling through CD2 and other costimulatory molecules also involves the actin cytoskeleton (52–55, 209–211). Microclusters of CD28 form concomitantly with TCR microclusters and then segregate into their own domain outside of the cSMAC, but the role of F-actin in the formation and centralization of these microclusters is unknown. Somewhat surprisingly, CD28 microclusters only require the presence of ligand to form and centralize and will do so even in the absence of the CD28 cytoplasmic tail, though differences in the rates were not addressed (212). This suggests that CD28 microcluster formation is primarily the result of kinetic segregation. If so, F-actin dynamics could contribute to this process, as diagrammed in Figure 5. Regardless of the effects of F-actin on CD28 microclusters, several studies indicate that propagation of signals downstream of CD28 is dependent on F-actin dynamics. First, the F-actin-uncapping protein Rltpr, which interacts with CD28, is absolutely required for CD28 signaling. Rltpr-deficient mice mimic the phenotype of CD28 knockout mice, and Rlptr is required for the CD28-mediated focusing of PKCθ and Carma 1 within the central region of the IS (49). Second, Tan et al. showed in thymocytes that activation of Src kinases by acute inhibition of Csk recapitulates many early signaling events in the TCR signaling pathway, but does not allow elevation of intracellular Ca^2+^ or ERK phosphorylation (45). Intriguingly, this blockade in signaling could be overcome by perturbing the actin cytoskeleton or by stimulating CD28-mediated F-actin rearrangement. These data support a model in which cortical actin forms a functional barrier between active PLC-γ1 and its substrate, and engagement of CD28 remodels actin architecture to allow signaling to proceed. Finally, it has been shown that costimulatory signaling by CD28 can induce greater force on stimulatory surfaces than TCR triggering alone (presumably through regulation of the F-actin network). This additional force is not applied through CD28 itself. Instead, force is applied through the TCR, at least in the absence of integrin engagement (138). Perhaps CD28 costimulation can lead to greater forces on other mechanosensitive receptors, including integrins. This idea is consistent with the finding that CD80 and CD86 on DCs increase the strength of cell–cell interactions with a responding T cell (213).

CD28 interacts with the F-actin cross-linking protein filamin A (FLNa) through the PxxPP motif in the CD28 cytoplasmic tail and domains 10–12 of FLNa. FLNa is recruited to the IS in a CD28-dependent manner following TCR stimulation, and CD28 interaction with FLNa is required for T cell costimulation (214). FLNa is a large, rod-like protein that is composed of an N-terminal actin-binding domain and 24 Ig-like domains. Ig-like domains 1–15 are referred to as Rod 1, while domains 16–23 make up Rod 2, with the 24th domain allowing for homodimerization. In addition to binding actin, FLNa is a prolific scaffolding protein with over 90 known binding partners, including intracellular signaling molecules, receptors, ion channels, transcription factors, and adhesion proteins (215, 216). Critically, many of these interactions can be regulated through the application of either external or internally generated force. Cryptic binding sites in the compact Rod 2 domain are exposed, and binding sites in the normal state are abolished following the application of force. This could occur through stretching of the Rod 2 domain under conditions where the F-actin network is under stress (216–218). This is particularly relevant at the IS, where the robust F-actin flow is likely to apply considerable stress to the network and may represent an important force-dependent aspect of CD28-mediated costimulation (Figure 5). Indeed, the mechanical regulation of FLNa-binding partners sets up several potential signaling mechanisms. Molecules that are recruited under force could allow for localized signal activation and signal amplification. Conversely, molecules that are released following force application could act as soluble signaling factors, exerting their function on areas distant from the IS. FLNa has been shown to be important for PKCθ recruitment to, and NF-κB-inducing kinase (NIK) activation at, the IS. Interestingly, while NIK is constitutively associated with FLNa, interaction with PKCθ requires CD3/CD28 signaling. Thus, it will be interesting to see if FLNa-mediated CD28 recruitment of PKCθ is force dependent.

### Force-Based Activation of Other Mechanosensitive Molecules at the IS

The preceding sections address force-induced activation of surface expressed receptors and their ligands, but F-actin-generated forces affect cytoplasmic molecules as well. One prime example is CasL, a lymphocyte-specific member of the Crk-associated substrate (Cas) family of proteins. Members of the Cas family contain a highly conserved Src kinase substrate domain with multiple phosphorylatable YxxP motifs. For the non-hematopoietic isoform p130Cas, it has been shown that these motifs are exposed by mechanical stretching of the protein (219, 220). Stretching of p130Cas and exposure/phosphorylation of the Crk-binding site is dependent on integrin binding to an immobilized substrate. In T cells, phosphorylation of CasL allows binding to Crk and the associated GEF C3G, leading to the activation of the small GTPase Rap1. Since Rap1 activation induces the recruitment of talin and the affinity maturation of multiple integrins, it seems likely that CasL functions in a positive feedback loop linking mechanical forces on engaged integrins to additional integrin activation.

Two independent studies have demonstrated that myosin contractility is required for maximal CasL phosphorylation, though in both studies there was significant CasL phosphorylation left at the IS following myosin inhibition (221, 222). Interestingly, phosphorylation of Cas within the substrate domain is largely independent of myosin contractility, but completely dependent on F-actin polymerization (150). It is therefore likely that even in the absence of myosin contractility, continued polymerization-driven F-actin flow at the IS provides sufficient force to drive stretch-dependent Cas/CasL activation.

### Cytoskeletal Forces May Create Signaling Rich and Poor Zones at the IS

Different mechanosensitive receptors can apply varying amounts of force on their ligands (223), and the maximum amount of force that can be applied is only as strong as the weakest link in the complex that links ligand to receptor and receptor to the actin network (199). Because of this, the force that allows for the greatest signaling for each receptor could be very different depending on the strength of these varying molecular interactions. In terms of the IS, TCR–pMHC catch-bonds can withstand a maximum force of roughly 10 pN. Unfolding of domain 20 in the CD28-binding protein FLNa occurs with the application of roughly 15 pN (224), while the integrin–ligand bonds are capable of withstanding maximum force of 30 pN (111, 194). Even the linkage of vinculin to the talin rod domains has a maximum force (~25 pN) that can be applied before unbinding occurs, since the talin helices become unstable (225). Since we have shown that the F-actin network slows as it moves toward the center of the IS, peak force is likely to decrease concomitantly [this assumes that force is directly proportional to the rate of F-actin flow, though reality may be more complex (199)]. If so, then signaling from force-resistant molecules would be initiated and sustained in regions of high and moderate F-actin dynamics (i.e., dSMAC and pSMAC regions), while molecular interactions with low force resistance would only occur in areas of moderate F-actin dynamics (i.e., the pSMAC region). This could set up intrinsic areas of maximal signaling for each receptor as microclusters form and traverse the IS. Additionally, since F-actin dynamics are poor or non-existent in the cSMAC, and the cSMAC represents an area of low force generation during the stable phase of IS formation (138), this region should not support force-dependent signaling. In keeping with this model, TCR microclusters retain their phosphorylation in the pSMAC, but become poorly phosphorylated in the cSMAC (77). Thus, the distribution of F-actin-generated forces at the IS may serve both to initiate signaling and to limit ongoing signaling by sweeping microclusters into areas of poor F-actin dynamics. In keeping with this, limiting the centralization of microclusters and maintaining them in the peripheral F-actin-rich compartments of the IS enhance microcluster lifetime and signaling (77). Additionally, for molecules that engage in catch-bond molecular interactions, the bond strength and half-life may also be regulated across the IS radius, with stronger interactions occurring in areas of higher F-actin-generated force, as long as that force is not above the rupture force. To really understand how changing actin rates and actin-generated forces affect T cell signaling differentially across the IS, careful measurements of traction forces on different molecules across the IS radius will be required.

## The Contribution of the Dendritic Cell Cytoskeleton to T Cell Priming

Although a great deal is known about the mechanisms through which T cell signaling affects actin dynamics on the T cell side of the IS, and *vice versa*, much less is known about the APC side of the synapse. The APC has long been assumed to be a passive partner in IS organization, and until recently, little attention has been paid to the possible contribution of the APC cytoskeleton. However, recent evidence indicates that at least for DCs, the F-actin network plays a critical role in regulating IS-associated signaling events leading to T cell activation.

### DCs Form Barriers to Lateral Diffusion and Control Synaptic Patterns

One area where the DC cytoskeleton is likely to play an active role is in regulating IS formation and structure (226). T cells responding to B cells or stimulatory supported lipid bilayers form the classical mature synapse with a characteristic annular pSMAC and cSMAC pattern (227, 228). This shows that in the absence of barriers to ligand mobility, TCR and LFA-1 microclusters will be driven toward the IS center in a T cell autonomous fashion. The DC/T cell IS lacks this annular pattern and is instead characterized by multiple patches of protein segregation. Even at late time points, there is no central accumulation of CD3 (229). Therefore, it is highly likely that the DC forms barriers to the free diffusion of T cell ligands that are either cytoskeletal or topological in nature. Importantly, these two possibilities are not mutually exclusive and the lateral mobility of some proteins could be regulated through linkage to the DC actin cytoskeleton, while others could be restricted by topological barriers. In either case, the DC actin cytoskeleton would play a crucial role in defining and maintaining these diffusive barriers. The existence of barriers at the DC/T cell IS has important implications for the mechanical regulation of signaling. As detailed below, we found that the T cell actin cytoskeleton activates mechanosensitive molecules, such as integrins, by applying force to the receptor–ligand bond, while barriers to diffusion provided by the DC cytoskeleton provide a retentive counter force on the ligand, thereby increasing tension at the molecular level. Through a similar mechanism, regulation of ligand mobility could prevent microcluster centralization and deactivation, thereby enhancing T cell activation. Just as upregulation of T cell ligands enhances T cell priming by mature DCs, control of molecular mobility would serve as a second mechanism through which DCs could modulate their T cell stimulatory capacity.

### The DC Cytoskeleton Plays a Critical Role in T Cell Priming

The DC F-actin network has been observed to polarize toward a cognate T cell in an MHC-dependent manner (230), and treatment of DCs with actin depolymerizing agents impairs the DCs ability to prime T cell responses (231). Polarization of the DC actin network is mediated by Rho-family GTPases and is required for proper conjugate formation, IL-2 production, and T cell proliferation (232–234). Similarly, the ARP2/3 complex activator WASp promotes the maintenance of T cell–DC interactions; WASp-deficient DCs exhibit fewer and shorter-lived contacts with cognate T cells, and a diminished ability to prime T cell proliferation (235, 236). Although it is clear that the F-actin network on the DC side of the synapse plays a key role in T cell conjugate formation and T cell activation, how changes in the DC cytoskeleton are regulated, and their role in IS-mediated signal transduction are open questions in the field.

### Maturation-Associated Changes in the DC Actin Network

Following recognition of danger signals through pattern recognition receptors, DCs undergo a maturation process that increases their stimulatory potential as APCs. Maturation is associated with an increase in F-actin content and increased plasma membrane ruffling, as well as major changes in actin-regulatory proteins. The resulting cytoskeletal changes alter antigen uptake and migratory behavior and increase the stimulatory potential of DCs by modifying the F-actin network at the DC–IS. Among the actin-regulatory proteins that are upregulated during maturation are the actin bundling protein fascin, which is greatly increased in expression (237, 238), the actin-severing protein cofilin, which is activated by dephosphorylation (239), and the actin-binding protein moesin, which is increased both by enhanced expression and phosphorylation-dependent activation (240). Interestingly, there is evidence that fascin and cofilin can work together to remodel the F-actin network (241). Moreover, fascin polarizes to the site of T cell engagement on DCs (231), and we have observed that T cells preferentially bind to pre-formed moesin-rich regions (240). It is important to point out that in addition to changes in proteins such as fascin, cofilin, and moesin that directly bind to actin filaments, DC maturation is associated with changes in regulatory molecules that control actin dynamics. For example, the activation of the Rho-family GTPase CDC42 is diminished during DC maturation (242). Similarly, the semaphorin receptor plexin-A1 is upregulated during maturation and is recruited to the DC–IS, where it mediates Rho activation, F-actin polarization, and T cell activation (234, 243). Going forward, it will be important to define how these changes in cytoskeletal proteins and their regulators impact DC–IS function. Likely mechanisms include physical reorganization of the DC membrane and generation of cytoskeletal tethers or corrals that limit the lateral mobility of T cell ligands.

### Regulated Mobility of T Cell Ligands on the DC Surface

Consistent with the idea that activation of T cell surface receptors is force dependent, *in vitro* analysis shows that modulation of ligand mobility can influence T cell activation (244). Though comparatively few studies have addressed ligand mobility on the DC surface, there is already evidence that ligand mobility is regulated in ways that are important for T cell priming.

#### Control of MHC Lateral Mobility

The strongest evidence that ligand mobility is important for TCR triggering comes from studies in which diffusion of an αCD3 antibody in stimulatory bilayers is limited by a physical barrier. Under these conditions, TCR microclusters are trapped in the periphery of the IS, resulting in increased microcluster phosphorylation and cellular activation (77). Interestingly, limiting the forward mobility of TCR microclusters causes local deformation of the F-actin network (6, 74, 245), suggesting that molecular forces between the TCR and the viscoelastic actin network are increased.

Though MHC class II lateral mobility is not constrained by the F-actin network in B cells or DCs (57, 240), the lateral mobility of MHC class I is restricted by the actin cytoskeleton (246–248). It is worth noting that many of the studies documenting TCR catch-bonds were done using the OTI or 2C CD8 TCR transgenic models, suggesting the possibility that TCR mechanotransduction and control of MHC lateral mobility may be more important in MHCI/TCR than MHCII/TCR interactions. It will be interesting to examine the lateral mobility of MHCI in professional APCs and to ask if the control of MHCI lateral mobility correlates with TCR triggering.

#### Control of Integrin Ligand Lateral Mobility

As mechanosenstive proteins, integrins respond to the physical properties of their ligand-binding environment. In fact, integrin-mediated cell spreading does not occur unless the ligand can withstand roughly 40 pN of applied force (223). In line with this idea, stiffness of the extracellular matrix correlates with outside in signaling (249), and surface immobilization of ICAM-1 is required for TCR-induced LFA-1 conformational change (193, 250). Importantly, the lateral mobility of ICAM-1 can have dramatic effects on immune cell function. In particular, NK cells adhere firmly to target cells in which ICAM-1 lateral mobility is low, and increasing ICAM-1 mobility decreases the efficiency of conjugate formation and lytic granule polarization (251). This suggests that restriction of the lateral mobility of integrin ligands increases tension on the ICAM-1/LFA-1 bond. In endothelial cells, members of the actinin and ERM family of actin-binding proteins limit the lateral mobility of ICAM-1 through interactions with a concerted polybasic region on the ICAM-1 cytoplasmic tail. Importantly, the constrained lateral mobility of ICAM-1 greatly increases the efficiency of T cell diapedesis, suggesting that this is a critical determining factor for LFA-1 adhesiveness (252, 253). We have recently shown that during DC maturation, ICAM-1 undergoes a specific decrease in lateral mobility mediated by interactions with moesin and α-actinin, which are upregulated and activated in response to inflammatory stimuli (240). This decrease in ICAM-1 lateral mobility enhances conjugate formation and LFA-1 affinity maturation, and ultimately contributes to T cell priming. This evidence indicates that DC maturation is associated with biophysical changes that constrain ligand mobility and promote mechanical integrin activation.

#### Control of CD80/86 Lateral Mobility

Constrained lateral mobility of the CD28 ligands CD80 and CD86 on the APC surface may also be important for APC function. Consistent with this idea, the cytoplasmic tails of CD80 and CD86 are essential for their costimulatory activity and mediate the separation of CD28 microclusters from TCR microclusters (254). These tails contain a highly conserved poly-basic motif that mediates protein clustering and cytoskeletal interactions (255, 256). This motif resembles known ERM-binding sites in other proteins, including ICAM-1 (253, 257). Given the importance of F-actin linkage and reorganization to CD28 function, it will be interesting to see if DCs can modulate costimulatory signals by regulating the lateral mobility of CD80 and 86.

### T Cells and DCs Coordinately Regulate the Activation of LFA-1 at the IS

Although there is increasing evidence that the TCR, CD28, and integrins can be activated by application of external forces on individual receptors, it is critically important to know if the molecular forces generated internally at the IS can drive these same activation pathways. We have recently shown that T cell actin retrograde flow drives conformational change of the integrin LFA-1 into the high affinity form, as well as its accumulation and organization at the IS (193). In a reciprocal process, DCs actively limit ICAM-1 lateral mobility on the plasma membrane through upregulation of the proteins moesin and α-actinin-1 (240). The limitation on ICAM-1 lateral mobility opposes the forces applied by the T cell actin cytoskeleton, thus enhancing tension and promoting LFA-1 affinity maturation, T cell adhesion, and priming. Thus, the F-actin networks in the T cell and the interacting DC work in concert to efficiently activate the integrin LFA-1. Interestingly, coordinated regulation of force application on LFA-1 and constrained ICAM-1/ligand lateral mobility is also at play during T cell migration on and diapedesis through inflamed endothelium (253, 258, 259). The theme that appears is that T cell adhesion to either APCs or endothelial cells, whether that is brought on by antigen recognition or exposure to chemokine, involves both a concerted change in the T cell and a reciprocal change in the interacting partner. Thus, for mechanosensitive molecules involved in T cell function, the physical properties of the ligand on the surface of interacting cells must be considered along with the forces applied to the receptor itself.

## Future Directions for Basic and Translational Research

Our understanding of the mechanobiology associated with signaling events at the IS is in its infancy. In particular, we lack concrete information on how the relevant forces are generated at the IS, which molecules act as true mechanosensors, and how the function of these molecules is coordinated to tune the immune response. Pioneering studies using biophysical approaches, such as modulation of substrate stiffness, physical manipulation using micropipettes, and traction force measurements, have all been informative (81, 138, 260), and there is much work yet to do in this arena.

To relate these biophysical measurements to T cell signaling, we also need to develop additional probes for protein conformation and activation state. For example, antibodies specific for integrin activation intermediates have been quite valuable (175, 193), as have antibodies that detect stretching of Cas (150, 222). Conformational probes for integrins and Cas family members have been successful because these proteins undergo large scale, concerted, changes as part of their regulated function. Given the paucity of robust conformational changes that are detectible in the ectodomains of the TCR, it is unsurprising that similar reagents do not exist for the study of TCR mechanosensing. One conformation-specific antibody has been reported for the TCR, though this epitope is exposed upon pMHC binding even in force-free conditions (131). Building upon work showing that agonist mAbs bind to the membrane-distal CD3ϵ lobe and exert torque on the CD3 complex (136), it may be possible to ask if cytoskeletal forces on TCRs engaged to pMHC have a similar effect by measuring changes in binding of anti-CD3ϵ Fabs in the presence or absence of F-actin dynamics. In any case, it seems likely that analysis of TCR force sensing will continue to be challenging, and that novel tools that detect changes transmitted across the cell membrane will be needed.

One caveat to the use of conformation-specific antibodies is that they often induce or stabilize the conformations they detect. This considerably limits their use, especially in live cell experiments. Thus, future studies are likely to require fluorescent tension biosensors for live cell microscopy similar to those that have been used to detect force on vinculin (261). This technology is evolving rapidly (262, 263). FRET-based biosensors have already been used to detect activation of VLA-4 in migrating T cells (263), and similar biosensors have been used to create integrin ligands that register force applied by spreading cells (264, 265). Another ligand-based approach involves a tension gauge tether consisting of a piece of double stranded DNA attached to an integrin ligand on one strand and to the substratum on the other such that the DNA strands separate with applied force, which can be varied over a range of 12–56 pN (223). Related ligand-based force sensors could be extremely useful in measuring the forces applied to specific receptor–ligand pairs, and the effects of cytoskeletal perturbations on these forces.

As a closing note, it is worth considering how new knowledge about the mechanobiology of T cell signaling could influence immunotherapy. Recent clinical successes have generated great interest in T cell-based immunotherapies for cancer and infection (266, 267). Although many approaches exist for adoptive T cell therapy, a common first step involves the *ex vivo* expansion of T cell subsets, typically through the use of stimulatory beads. Much thought has gone into optimizing the antibody cocktails used for bead coating, but relatively little attention has been paid to their physical properties. Yet, these properties are likely to be highly significant. For example, most stimulatory magnetic beads commercially available for T cell activation are ~4 μm in diameter; yet, human peripheral blood T cells engaging APCs readily spread to a diameter of 12 μm and resting blasts can spread to over 20 μm. A bead that is not large enough to induce robust spreading is not likely to induce the symmetric actin flow associated with mechanical signaling. Thus, beads with a larger surface area may enhance T cell activation and expansion. Another key parameter is the rigidity of the stimulatory surface. Human T cells reportedly respond better on softer surfaces, with increased IL-2 secretion, proliferation, and effector function increasing upon decreased cell surface rigidity (81). Interestingly, the substrate rigidity range used in this study was 100–1000 kPa, several orders of magnitude above physiological values for soft lymphoid tissues. Further research aimed at optimizing surface area and rigidity may prove valuable in creating artificial APCs for clinical applications.

## Author Contributions

WC and JB reviewed and interpreted the literature, drafted and revised the manuscript, and approved the final version for publication. They both agree to be accountable for all aspects of the work in ensuring that questions related to the accuracy or integrity of any part of the work are appropriately investigated and resolved.

## Conflict of Interest Statement

The authors declare that the research was conducted in the absence of any commercial or financial relationships that could be construed as a potential conflict of interest.
